# Expression of the H19 Oncofetal Gene in Premalignant Lesions of Cervical Cancer: A Potential Targeting Approach for Development of Nonsurgical Treatment of High-Risk Lesions

**DOI:** 10.1155/2013/137509

**Published:** 2013-07-31

**Authors:** Tomer Feigenberg, Ofer N. Gofrit, Galina Pizov, Avraham Hochberg, Abraham Benshushan

**Affiliations:** ^1^Department of Obstetrics and Gynecology, Division of Gynecologic Oncology, University of Toronto, Princess Margaret Cancer Center, 610 University, Avenue M-700, Toronto, ON, Canada M5T 2M9; ^2^Department of Urology, The Hadassah Ein-Kerem Medical Center, The Hebrew University, 91120 Jerusalem, Israel; ^3^Department of Pathology, The Hadassah Ein-Kerem Medical Center, The Hebrew University, 91120 Jerusalem, Israel; ^4^Department of Biological Chemistry, The Alexander Silberman Institute of Life Sciences, The Hebrew University of Jerusalem, The Edmond J. Safra Campus, Givat Ram, 91904 Jerusalem, Israel; ^5^Department of Obstetrics and Gynecology, The Hadassah Ein-Kerem Medical Center, The Hebrew University, 91120 Jerusalem, Israel

## Abstract

*Background*. Recent data suggest a role for H19 gene in promoting cancer transformation and progression. Cervical cancer, progresses from high-grade lesions (CIN3). At present, it is unclear if CIN lesions express H19. *Objectives*. To determine H19 expression in patient samples of CIN3 as well as the ability of a construct in which the promoter from the H19 gene drives expression of the diphtheria toxin A chain (DTA) to inhibit cervical cancer cell growth *in vitro*. *Methods*. H19 transcript levels were evaluated on 10 biopsies of CIN3 using *in situ* hybridization. PCR was used to examine H19 expression in cervical cancer cell lines and in two samples from a patient with cervical carcinoma. Cell lines were transfected with H19-DTA to determine its impact on cell number. *Results*. H19 gene was expressed in the area of CIN3 in 9 out of 10 samples. RT-PCR indicated expression of H19 in cervical cancer samples and in one of the three cell lines examined. Transfection of all cell lines with H19-DTA vector resulted in inhibited cell growth. *Conclusions*. H19 is expressed in the majority of CIN3 samples. These results suggest that most CIN3 lesions could be targeted by H19-DTA. Further *in vivo* preclinical studies are thus warranted.

## 1. Introduction

Carcinoma of the uterine cervix is the second most common cancer worldwide. Every year, cervical cancer is diagnosed in about 500,000 women globally and is responsible for more than 280,000 deaths. While most cases of cervical cancer are diagnosed in developing countries, the USA National Institute of Health estimated that in the USA during 2010, 12,200 new cases of cervical cancer would be diagnosed and over 4000 women would die from this disease.

The primary events leading to cervical dysplasia and carcinogenesis are most commonly related to infection with the human papilloma virus (HPV). Infection with HPV is diagnosed in as many as 99% of the women diagnosed with squamous cervical carcinoma cancer of the uterus. HPV is a double-stranded DNA virus that affects the growth and differentiation of cervical epithelial cells by interaction between the envelope proteins E6, E7 and tumor suppressor genes such as P53 and the retinoblastoma gene (Rb). Infection of cervical epithelial cells with the HPV causes the formation of early premalignant lesions, referred to as cervical intraepithelial neoplasia 1 (CIN1). In most cases, the cellular immune system eliminates these cells, resulting in a return to a normal epithelium. However, in some cases, these low-grade lesions progress to high-grade lesions (CIN3) and frank cervical cancer. It is possible that this oncogenic process is related to an abnormal viral cycle, in which the HPV cannot complete its normal infective cycle; instead, the viral DNA becomes incorporated into the host cell genome. If this occurs, there is a greater chance of malignant transformation [1]. The current treatment for high-grade dysplasia of the uterine cervix is surgical excision of the involved cervix by performing conization, which increases the risk of future premature deliveries. At present, there are no effective medical approaches for the treatment of CIN3 lesions. 

 An attractive approach to human cancer gene therapy is to exploit unique genetic and epigenetic alterations in tumor cells for targeting the expression of toxic genes. Cytogenetic and epigenetic alterations have been associated with CIN3 and squamous cervical cancer, most notably a gain in chromosomal region 3q26 [2, 3]. Two recent papers have also reported abnormal expression of the H19 oncofetal gene due to deletions and/or abnormal imprinting in cervical cancer specimens [4, 5]. H19 is a paternally imprinted, maternally expressed gene that encodes RNA that acts as a “riboregulator,” having no protein product [6]. H19 is expressed at substantial levels in several different human tumor types [7–10], but only minimally or not at all in normal adult tissues [7, 11]. The function of the H19 gene is not known; however, recent data suggest a role in cancer progression, angiogenesis, and metastasis [12, 13]. The human H19 gene lies within 200 kb downstream of the paternally expressed IGF2 gene at 11p.15.5, and shared enhancers downstream of H19 coordinate transcription of both genes [14]. 

 We have used the transcriptional regulatory sequences of the H19 gene to drive expression of a toxic gene, diphtheria toxin A chain (DTA), which has suitable properties for achieving efficacious cancer cell killing [15, 16]. The therapeutic potential of the H19-DTA vector was tested in a rat animal tumor model for colorectal liver metastases, showing tumor growth inhibition in the H19-DTA-treated group as compared to the control group [17]. H19-DTA was also found to be effective in ovarian carcinoma cell lines and in a heterotopic animal model for ovarian cancer [18].

 In the present study, we investigated H19 expression in clinical samples of CIN3 as an initial step in determining the potential of H19-DTA or other H19 targeting approaches in the medical treatment of these lesions. Since premalignant human-derived cell lines or valid animal models for premalignant cervical lesions are not currently available, we examined the ability of the H19-DTA construct to inhibit cell growth of available cervical cancer cell lines.

## 2. Materials and Methods

Institutional review board approval (from the Hadassah-Hebrew University Medical Center, Jerusalem Israel, Ethics Committee) was obtained prior to initiating the experiments.

### 2.1. Cell Culture

The human-derived cervical carcinoma cell lines (HeLa, SW756, and CaSky) used in this study were obtained from the American-Type Culture Collection (ATCC). Cells were maintained in DMEM-F12 (1 : 1) medium containing 10% fetal calf serum at 37°C in a humidified 5% CO_2_ environment. 

### 2.2. *In Situ* Hybridization (ISH) for H19

The expression of H19 was determined by *in situ* hybridization in formalin-fixed paraffin-embedded tissues from 10 patients diagnosed with CIN3. Paraffin sections were rehydrated through a series of alcohols, followed by a wash in 0.9% NaCl and then in PBS. Basic proteins were removed by incubation in 0.1 N HCl at room temperature for 15 min. The sections were then washed in distilled water, treated with 10 *μ*g/mL proteinase K (Sigma, Poole, Dorset, UK) in 50 mM Tris, 5 mM EDTA for 30 min at 37°C, and rinsed in 4% paraformaldehyde/PBS. The sections were rinsed in PBS and acetylated for 10 min in fresh acetic anhydrate diluted 1/400 in 0.1 M triethanolamine (Sigma) at pH 8.0. The slides were rinsed in PBS for 5 min in 0.9% NaCl for another 5 min, dehydrated, and air dried before hybridization. 

The hybridization buffer contained 50% deionized formamide, 0.3 M NaCl, 20 mM Tris-HCl (pH 7.4), 5 mM EDTA, 10 mM NaH_2_PO_4_ (pH 8.0), 10% dextran sulphate, 1X Denhardt's solution, and 0.5 *μ*g/mL yeast tRNA. Each section was covered with 30 *μ*L of the hybridization solution containing 30–100 ng of DIG-labeled RNA probe. Digoxigenin-labeled H19 RNA transcripts were produced by labeling with DIG-11-UTP by SP6, T3, or T7 RNA polymerase in an *in vitro* transcription reaction (Boehringer, Mannheim, Germany) as described previously [19]. The sections were covered with siliconized coverslips. Hybridization was performed at 48–52°C for 12–24 hours in a humidified chamber. After hybridization, coverslips were gently removed in 5X saline sodium citrate at 50°C for 30 min. Subsequently, tissues were subjected to a stringent wash at 60°C in 50% deionized formamide, 2X saline sodium citrate for 20 min. Following a double rinse for 10 min in washing buffer (0.4 M NaCl, 10 mM Tris-HCl, and 5 mM EDTA), treatment with RNase A (20 *μ*g/mL) was performed for 30 min at 37°C. The sections were then equilibrated in buffer 1 (100 mM Tris-HCl and 150 mM NaCl, pH 7.5) at room temperature and incubated in 1/50 whole sheep's serum for 30 min (to avoid nonspecific cross-reactions of the primary antibody). Incubation with the antidigoxigenin antibody (Boehringer) diluted 1/1000 in buffer 1 was performed for 2 hours at room temperature. After two washes in buffer 1, the sections were equilibrated in buffer 2 (100 mM Tris-HCl, 100 mM NaCl, and 50 mM MgCl_2_, pH 9.5) for 2 min and then with freshly prepared color substrate solution containing nitroblue tetrazolium salt (340 *μ*g/mL), 5-bromo-4-chloro-3-indolyl, phosphate toluidine salt (170 *μ*g/mL), and levamisole (1 mM) in buffer 2. The slides were placed in a humid chamber and allowed to develop in the dark for 12–14 hours at room temperature. The reaction was stopped in buffer 3 (10 mM Tris-HCl and 1 mM EDTA, pH 7.4) for 5 min. Finally, the sections were counterstained with 3% Gimsa stain, quickly rehydrated, and mounted in ethelan. Controls for the specificity of the ISH included RNase A pretreatment of sections; hybridization with a sense RNA probe and hybridization with hybridization buffer without the probe. A section of bladder carcinoma, which expresses H19, was used as a positive control.

### 2.3. Determination of H19 RNA by PCR

Total RNA was extracted from samples of cervical carcinoma obtained from a patient diagnosed with stage IIb cervical cancer and from HeLa, SW756, and CaSky cells using RNA STAT-60TM Total RNA/mRNA isolation reagent (Tel-Test, Inc. Friendswood, TX, USA), according to the manufacturer's instructions. The RNA was treated with RNase-free DNase I (Roche Diagnostics GmbH, Mannheim) to eliminate genomic DNA. cDNA was synthesized from 2 *μ*g total RNA in a 20 *μ*L reaction volume by 10 ng/*μ*L of oligo (dT)15 primer (Roche) and 10 units/*μ*L M-MLV Reverse Transcriptase (GibcoBRL, UK) according to the manufacturer's instructions. An aliquot (200 ng) of cDNA was amplified by PCR. The PCR reactions were carried out in a 25 *μ*L volume containing 6 ng/*μ*L of each of the forward and the reverse primers, dNTPs, and 0.05 units/*μ*L of Taq polymerase (TaKaRa Biomedicals, Japan) according to the manufacturer's instructions. The primer sequences used to amplify the human H19 transcript were (5′-ACTGGAGACTAGGGAGGTCTCTAGCA) forward and (5′-GCTGTGTGGGTCTGCTCTTTCAAGATG) reverse. The PCR reaction was carried out for 29 cycles (98°C for 15 sec, 58°C for 30 sec, and 72°C for 40 sec) and finally at 72°C for 5 min. The products of the PCR reaction were run on 2% agarose in TAE electrophoresis running buffer (40 mM Tris acetate and 2 mM EDTA, pH 8.5), stained by ethidium bromide, and visualized by UV.

### 2.4. Constructs

#### 2.4.1. Luc-1 Construct

The luciferase pGL3 basic vector (Promega, Madison, USA) which lacks both promoter and enhancer sequences was used as a control construct and as a basis for the other constructs.

#### 2.4.2. Luc-H19 Construct

The Luc-H19 construct contains the luciferase reporter gene under the control of the human H19 promoter region from nucleotide −818 to +14, and was prepared as previously described [20]. 

#### 2.4.3. H19-DTA Construct

The Luc-H19 plasmid was digested with Xba I and Nco I, and the insert containing the luciferase gene (*luc*) was replaced by the diphtheria toxin A chain (*DTA*) coding region to yield the DTA-H19 construct. Large-scale preparations of the plasmids were performed using the EndoFree Plasmid Mega kit (Qiagen, Germany). All plasmids were modified by replacing the ampicillin-resistant gene with the Kanamycin resistant gene.

### 2.5. *In Vitro* Transfection and Luciferase Assay

A total of 10^5^ cells per well were plated in twelve-well Nunc multidishes (30 mm). Transient transfections were carried out using the JetPEI cationic polymer transfection reagent (mean molecular weight of 22 kDa; Polyplus, Illkirch, France) according to the manufacturer's instructions using 2 *μ*g of DNA and 3 *μ*L of JetPEI solution to obtain an N/P ratio of 5. Transfection experiments were stopped after 48 h, and reporter gene activity was assessed. Luciferase activity was measured using the Promega kit “Luciferase Assay System” (E-1500; Promega, Madison, USA). Light output was detected using a Lumac Biocounter apparatus. Protein content was measured by the Bio-Rad (Hercules, CA, USA) protein assay reagent, and the results were expressed as light units/*μ*g protein. LucSV40 (Luc-4) was used as a reference for maximal luciferase activity, as it contains the SV40 promoter and enhancer, while Luc-1 lacking regulatory sequences was used as a negative control to determine the basal non-specific luciferase expression, which was found to be negligible. All experiments were conducted in triplicate and repeated 3 times. The results represent the mean value of all of the experiments. In all of the transfection experiments, measured luciferase activity is expressed as a percentage of that observed after transfection with the positive control plasmid (Luc-4) alone, to allow normalization of luciferase activity.

## 3. Results

### 3.1. Expression of H19 RNA in CIN3 Lesions

**Figure 1:**
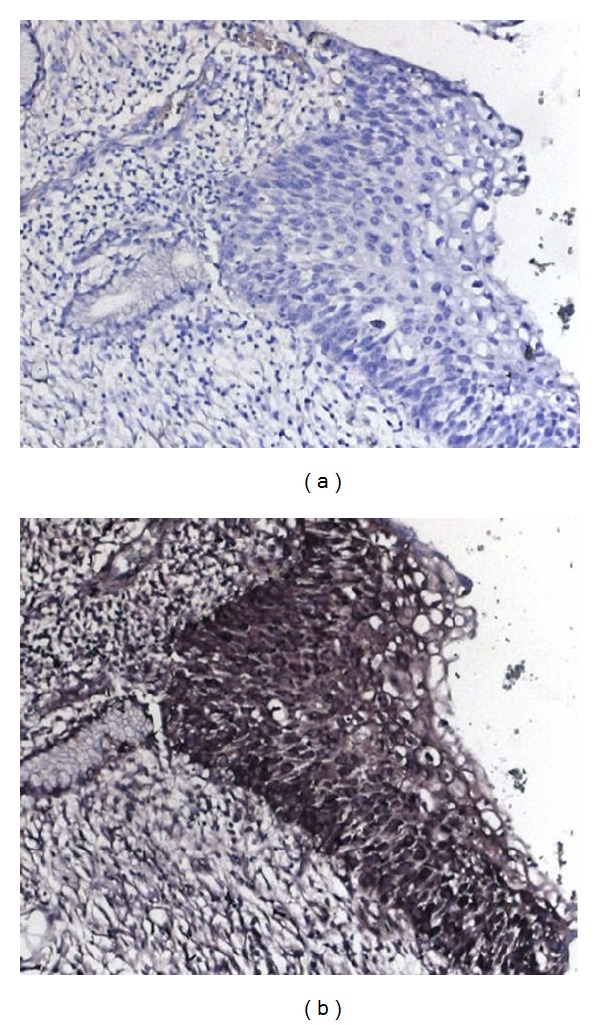
H19 transcripts in a slide from a patient diagnosed with CIN3 determined by ISH analysis (Gimza stain on the right).

To test the expression of H19 mRNA in premalignant cervical lesions, we collected formalin-fixed, paraffin-embedded slides from ten patients diagnosed with CIN3. ISH for H19 RNA was conducted as described in the Methods section. In each slide, the intensity of staining and area of staining were compared to a negative control and graded on a scale of 1–3, by a pathologist. The area of CIN3 that was positively stained was compared with the total area of CIN3 in each slide. The area of staining was scored 1 if less than 33% of the area was positive, 2 for an area of 33%–66%, and 3 for an area larger than 66%.  Table 1 presents the results of ISH for the 10 samples. All samples but one were positive.  Figure 1 shows a representative ISH image. Our results also demonstrate that, while areas of CIN3 express H19 as determined by positive DIG staining, surrounding normal cervical epithelium was not stained and thus expressed little or no H19.

### 3.2. The Level of H19 Transcript in Samples of Cervical Cancer and Human-Derived Cell Lines as Detected by PCR

**Figure 2:**
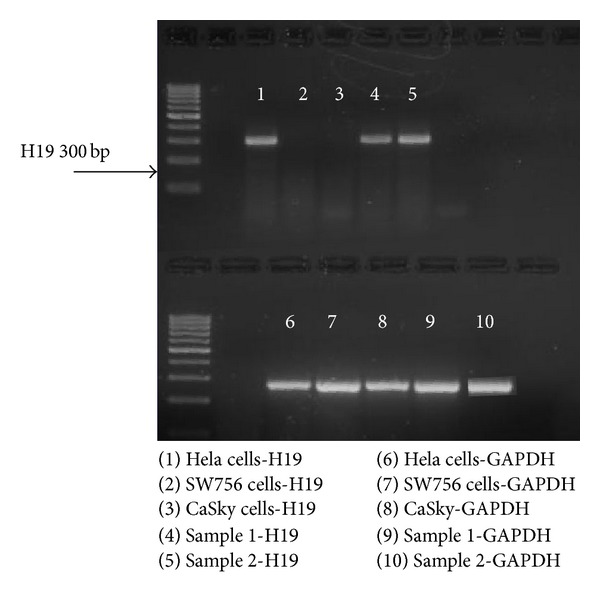
H19 transcript in RNA isolated from cell lines and patient samples determined by PCR.

The level of H19 transcripts in 3 different cervical cancer cell lines and in 2 samples of cervical cancer from a woman diagnosed with cervical cancer was determined by PCR (Figure 2). One cell line out of the three examined (HeLa) was positive for H19 RNA. The two surgical samples of cervical carcinoma were both positive. 

### 3.3. The Killing Effect of the H19-DTA Vector in Different Cell Lines

**Figure 3:**
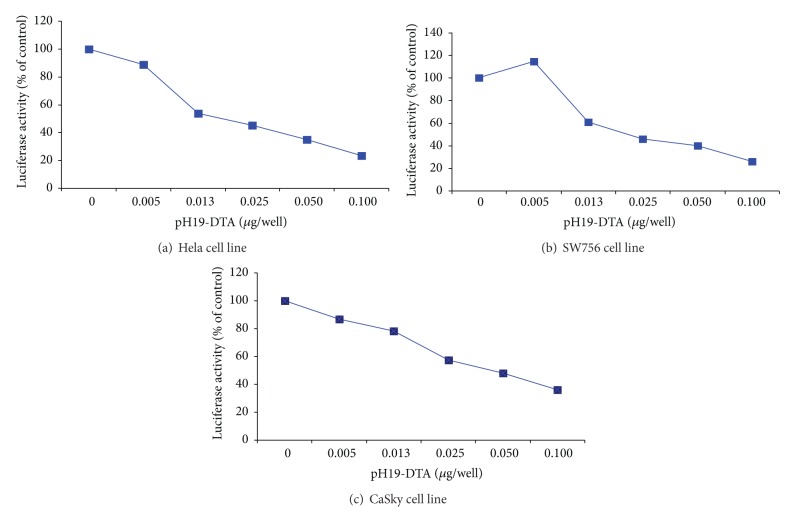
Relative reduction in luciferase activity induced by transfection of human cell lines with Luc-H19 plasmid.

Since it was not possible to test the impact of H19-DTA on CIN3 cells, the *in vitro* growth suppressive effects of the H19-DTA plasmid on 3 human derived cervical cancer cell lines: HeLa, CaSky and SW756, were examined. The cell lines were cotransfected with Luc-4, Luc-H19, and the indicated concentrations of H19-DTA (Figure 3). Luciferase activity was determined and compared with that of cells transfected with Luc-4 alone. The relative reduction of the luciferase activity in the cotransfected cells reflects the level of the H19-driven DTA expression and thus cell killing. We previously showed that cell lines resistant to diphtheria toxin [18] and nontransformed cells (primary fibroblast cell line IMR-90) [20] are minimally affected by the H19-DTA vector. In the present study, a significant decrease in luciferase activity was detected in all H19-DTA cotransfected cell lines. The relative reduction of the luciferase activity in the cotransfected cells was dependent on the dose of H19-DTA (Figure 3). 

## 4. Discussion

Using ISH, we show for the first time that H19 RNA is expressed in CIN3. Nine out of ten patient samples of CIN3 were positive for H19 transcript and this expression was specific to areas of CIN3. Areas of normal epithelium lacked detectable levels of H19 transcript. This finding suggests that expression of H19 RNA is an early phenomenon in cervical oncogenic transformation. At present, the only treatment available for CIN3 is surgical excision of the involved cervical tissue by conization, which may have detrimental effects on future pregnancies resulting in preterm birth. Since our group is involved in clinical trials evaluating the potential use of H19-DTA, one of the goals of the present study was to evaluate the potential for the use of this targeting vector for the treatment of CIN3. Unfortunately, premalignant cervical cancer cell lines are not available, and a valid animal model for CIN3 has not been established. Thus, to provide a further initial indication of the ability of H19 to direct DTA expression in pre- and malignant cervical cancer cells, we used established cervical cancer cell models. 

 Two previous studies examined the expression of H19 and IGF2 genes in samples from cervical cancer patients. The first study by Douc-Rasy et al. [5] examined methylation patterns of IGF2 and H19 genes in samples from patients diagnosed with widespread cervical cancer. According to that study, abnormal imprinting of those genes played a major role in the pathogenesis of the cancer in 58% of the samples assessed. In a later study by Kim et al. [4], different methylation patterns were examined in 32 tissue samples from women who were diagnosed with cervical cancer. They found an absence of IGF2 imprinting in 39% of the samples and an absence of H19 imprinting in 30.5% of the samples. In agreement with these reports, two samples from a patient diagnosed with cervical cancer were found to express H19 RNA. However, of the three human cervical cancer cell lines examined, only HeLa cells expressed H19 transcripts detectable by PCR. Despite this, all three cell lines were surprisingly inhibited by H19-DTA in a dose-dependent manner. The lack of correlation between H19 transcript levels and the efficacy of H19-DTA has been previously shown [18]. One possibility is that the amount of H19 transcript depends on its stability rather than on transcription rate as has been shown for smooth muscle tissue samples [21]. H19 RNA stability, therefore, may be largely controlled by local proteins that stabilize the RNA. It is possible that, despite high replication rate of the H19 gene that enables effective vector cell killing, due to low stability, the H19 RNA was not detected by PCR. Alternatively, there may be differences in methylation pattern between the H19-DTA vector and the H19 primers that were used for PCR. 

The successful development of anti-tumor gene therapy depends on the use of combined approaches aimed at targeted delivery and specific expression of effective anti-tumor agents. The H19-DTA vector exploits a tumor-selective promoter in conjunction with a cytotoxic gene to achieve targeted tumor cell destruction. The H19 promoter has been proven to be highly active in various tumor types and to show marginal or no activity in the surrounding normal tissue [19, 22]. It was previously shown that the H19-DTA constructs are able to selectively kill tumor cell lines and inhibit tumor growth both *in vitro* and *in vivo* [20, 23]. The choice of DTA as a toxin gene ensures not only high killing activity but also low toxicity since DTA protein released from lysed cells is not able to enter neighboring cells in the absence of the DTB fragment. This modality's efficacy in treating bladder and ovarian cancer are currently being evaluated in ongoing clinical phase I/II trials. Given the external accessibility of CIN3 lesions and the protracted time involved in transformation of these lesions to invasive carcinomas, H19-DTA may offer a potentially attractive alternative to surgical management of these lesions. This approach would offer the opportunity to resample the cervix after the treatment and to perform surgical excision if deemed necessary. Although the development of reliable animal models for CIN3 was beyond the scope and funding of the present study, the current findings support further assessment in preclinical models. 

In conclusion, we demonstrate for the first time that CIN3 samples express the H19 gene exclusively in areas of CIN3 within the cervical epithelium. We also show that a novel strategy for selective cervical cancer cell killing is effective *in vitro* for cervical cancer cells. Future preclinical studies are thus warranted to determine whether H19-DTA vector is effective as a medical treatment for eradication of CIN3.

## Figures and Tables

**Table 1:** The level of H19 RNA transcript in samples obtained from patients diagnosed with CIN 3 determined by ISH analysis.

Patient number	Intensity	Area
1	++	+++
2	++	+
3	+++	+++
4	++	+++
5	+++	+++
6	+++	+++
7	+++	+++
8	+	+
9	+++	+++
10	−	−

*Areas of surrounding normal cervical epithelium did not stain for H19 by ISH in all 10 samples.
